# Smartphone Apps for Mindfulness Interventions for Suicidality in Asian Youths: Literature Review

**DOI:** 10.2196/mhealth.8304

**Published:** 2018-06-07

**Authors:** Carol C Choo, André AD Burton

**Affiliations:** ^1^ James Cook University Singapore Singapore; ^2^ School of Psychology Curtin University Western Australia Australia

**Keywords:** suicidality, Asian youths, smartphone applications, mindfulness

## Abstract

**Background:**

The advent of mobile technology has ushered in an era in which smartphone apps can be used as interventions for suicidality.

**Objective:**

We aimed to review recent research that is relevant to smartphone apps that can be used for mindfulness interventions for suicidality in Asian youths.

**Methods:**

The inclusion criteria for this review were: papers published in peer-reviewed journals from 2007 to 2017 with usage of search terms (namely “smartphone application” and “mindfulness”) and screened by an experienced Asian clinician to be of clinical utility for mindfulness interventions for suicidality with Asian youths.

**Results:**

The initial search of databases yielded 375 results. Fourteen full text papers that fit the inclusion criteria were assessed for eligibility and 10 papers were included in the current review.

**Conclusions:**

This review highlighted the paucity of evidence-based and empirically validated research into effective smartphone apps that can be used for mindfulness interventions for suicidality with Asian youths.

## Introduction

Suicide rates increase with age from adolescence to young adulthood [1,2], with corresponding heightened rates of suicidal ideation and attempts [3]. Both Western and Asian studies have highlighted the prevalence of youth suicidality, and youth suicide rates have been rising faster compared to other age groups [4,5], with a peak in those between 15 and 24 years of age [3,6,7]. In a recent large study on Asian suicide attempters in Singapore, a prominent peak in suicide attempts over a 3-year period was observed in youths aged between 15-24 years, as compared to other age groups [4].

In recent decades, the advent of smartphone technologies has transformed the mode of delivery of psychological treatment [8] for patients suffering from chronic medical illnesses [9,10] and psychiatric illnesses [11], as well as their caregivers [12]. The demand for electronic health apps across the world is mirroring larger societal trends wherein consumer acceptance of technology has grown [13,14].

Common psychiatric illness such as depression are associated with high direct and indirect costs [15]. Psychiatric illnesses result in functional impairment, leading to lost wages and work impairment, with related personal, societal, and economic burdens [16,17]. Smartphone apps have the potential to reduce health care costs for treating psychiatric illnesses in Asia [18,19]. In comparison to Western countries, there is a shortage of mental health professionals in Asia, yet a high penetration of mobile phone usage throughout Asia [20]. Over 50% of the Asian population uses smartphones, with Singapore alone reporting that smartphone adoption rates far exceeded the population [21]. There is a critical need for comprehensive research to inform the development of evidence-based smartphone apps that can be made widely available for the public, to ameliorate symptoms and improve well-being in Asian populations.

As younger demographics are more likely to access online information related to mental health problems [22-24], mobile technologies can enhance patient-centered care for youths in an increasingly technology savvy society [25], highlighting a growing need to offer electronic interventions [26,27]. The evidence base for the use of smartphone apps has been demonstrated in many areas [28-33], and Internet-based interventions have been found to be efficacious for mental health issues [34] in young adults [23,26,35] to enhance support [36], help them to cope, and to aid in recovery [37,38]. Positive outcomes were shown in overall motivation [39] and with ethnically diverse populations [40]. Smartphone apps have been used to deliver therapies that are relevant to young adults, such as cognitive behavior therapy [10], addiction treatment [41], and virtual reality therapy [42]. These findings hold promise for mental health professionals who are not technical experts to develop smartphone apps as an alternative platform to deliver interventions, in view of the recent advances in technology [43]. However, it should not be assumed that smartphone apps delivering interventions demonstrated to be effective in Western cultures will be similarly effective in Asian cultures [44]. Cultural adaptations may be needed for Asian youths [45].

Some clinics in Australia have implemented conjunctive treatment modalities in guided programs such as Cognitive Behavioral Therapy and psychoeducation apps alongside face-to-face therapy sessions [40]. One example is the Dialectical Behavioral Therapy Coach, which was an app that was designed after extensive feedback from experts [46]. The app aimed at cultivating emotional regulation skills to change negative emotions [47]. The users gave ratings and identified the current emotion, after which users were directed to specific coaching [47].

Such developments are currently lacking in Asia. As aforementioned, it should not be assumed that interventions demonstrated to be effective in Western cultures will be similarly effective in Asian cultures, especially those concerning suicidality. Culture plays an important role in determining risk and protective factors for suicidality, which informs targeted assessment and intervention strategies [44]. Asian suicide attempters are more likely to overdose on prescribed and over-the-counter medications instead of using firearms in their suicide attempts [48], as compared to Western samples, and Asian suicide attempters also endorse different views on the lethality of suicide methods [49].

Mindfulness interventions have been used to treat various psychological problems such as anxiety and depression [50-52]. Mindfulness practice reduces psychological distress while optimizing psychological functioning among young adults [53] by enhancing positive affect and lowering negative affect [51]. Large scale empirical research investigating the evidence base for mindfulness interventions in Asian samples seems to have gained momentum in the last few years [52]. Depression is a common psychiatric illness in Asia. Asians suffering from depression often experience maladaptive ruminations [54] and would be suitable for mindfulness-based therapies, which have been shown to contribute to significant reductions in maladaptive rumination [55]. Furthermore, youths are often affected by problems including low self-esteem [56], poor weight control [57], eating problems [58], Internet addiction [59], and chronic diseases including dermatitis [60] and asthma [61]. Mindfulness-based therapy shows evidence in improving self-esteem [62], weight control [63,64], eating problems [65], Internet addiction [66], and chronic diseases in Asian youths [67], and holds promise for use with Asian youths to enhance their overall wellbeing and resilience and reduce their vulnerability to suicidality [45]. Recent studies have drawn links between resilience, suicidality [44,68], and mindfulness practice for Asian populations [45,68]. In Asia, the stigma related to mental illness and suicidality may hinder help-seeking behavior in youths. This issue further increases their vulnerability to suicide risk [4]. These at-risk youths might prefer to access community interventions such as self-help on electronic platforms delivered using smartphone apps [20] rather than face-to-face therapy. Such apps offer an alternative delivery medium that is also cost effective [53]. The accessibility of such apps may enhance our efforts in primary prevention, and mental health promotion, aligned with recent research in Singapore. A recent study in Singapore highlighted the need for mental health promotion to reduce stigma related to psychiatric illnesses and enhance psychological wellbeing [45]. Recent research indicates that preventative mental health care involves enhancing resilience and promoting protective factors, which includes mindfulness-based interventions for emotional regulation [44,45,53].

There are many smartphone apps currently available that are marketed as mindfulness apps. A search using the search term “mindfulness-based iPhone Applications” from November 2013 yielded 808 results. This number is consistent with earlier research informed by a search for “mindfulness” conducted on iTunes and Google Applications for mindfulness training [36]. Such apps were reviewed by experts. However, the utility among Asian youth consumers remains unclear. Widespread implementation of self-help mindfulness interventions could be premature without salient evidence and scientific scrutiny for use by the intended population [69]. Youths can be impressionable consumers, and principles of rigorous scientific enquiry should be applied to explore therapeutic benefits of such apps [70]. Unfortunately, the utility of such apps for suicidality in Asian youths remains largely unexamined. Research aimed at examining low-cost smartphone apps that are efficacious as a therapeutic tool for suicidality in Asian youths would add significantly to the current literature [71]. Considering the heightened suicide risk uncovered by recent research with Asian youths [4], and the need for early prevention [45], research is needed to explore alternative ways to deliver effective interventions that are also cost effective and easily accessible. The aim of this paper is to review research relating to the evidence base for smartphone apps that can be used for mindfulness intervention for suicidality in Asian youths.

## Methods

The inclusion criteria for this review were: publications in peer-reviewed journals from 2007 to 2017 with usage of search terms namely “smartphone application” and “mindfulness.” Databases included PSYCINFO, SCOPUS, Google Scholar, and PubMed. The papers were retrieved if they related to interventions via smartphone apps for mindfulness interventions. The structured proforma for evaluating eligibility for inclusion involved the following: recent papers that contain original work published in peer-reviewed journals after the year 2007; papers related to usage of a smartphone app by clinicians for therapeutic purposes and considered by an experienced Asian clinician to be of clinical utility with suicidal youths in Asia.

The focus was on recently published papers in peer-reviewed journals that fit the inclusion criteria and were relevant to smartphone apps that can be used for mindfulness intervention for suicidal youths in Asia. The main reason for the exclusion of articles was the fact that papers did not refer to the use of smartphone apps by clinicians for therapeutic purposes.

## Results

**Figure 1:**
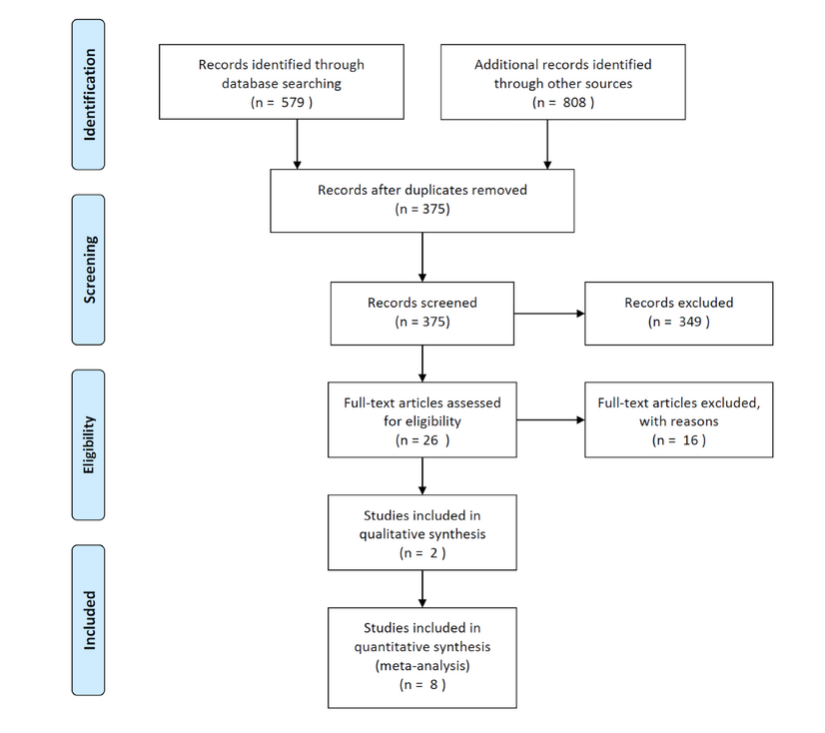
PRISMA flow diagram.

The *PSYCINFO* database was initially used to identify peer-reviewed papers with the inclusion criteria named above; this yielded 375 results when all search terms were used. From the original search results, the abstracts were screened, and 14 full text papers from peer-reviewed journals were then downloaded and assessed against the inclusion and exclusion criteria. See Figure 1 for the PRISMA flow chart [72]. Ten recent papers deemed to be suitable were included in the current review, with a focus on papers published in the last 5 years. The results of the review are presented in Multimedia Appendix 1. A review of papers in Multimedia Appendix 1 shows a lack of convincing evidence of the efficacy of smartphone apps that can be used for suicide interventions for Asian youths.

## Discussion

In summary, a review of papers presented in Multimedia Appendix 1 shows a lack of convincing evidence of the efficacy of smartphone apps that can be used for suicide interventions for Asian youths. A review of 15 randomized controlled trials, including 4 mindfulness-based interventions, indicates that mindfulness interventions significantly improve levels of mindfulness and depressive symptoms [69]. However, effect sizes were small to medium. There were high drop-out rates and few trials were adequately powered. Another recent study examined an app with Spanish features with a large sample size but employed statistical analyses that did not produce convincing evidence [73]. Other studies only reviewed apps [13,53,74] and did not test them on the intended users. Another study that examined mindfulness-based interventions found significant effects on well-being, stress, anxiety, depression, and mindfulness, but had a low representation of Asian youths [75]. Another study found significantly increased positive affect and decreased depression but no statistically significant difference in satisfaction with life or negative affect, and again this study had low representation of Asian youths in the sample [76]. A study on Chinese youths [77] found that an online mindfulness intervention had a significant effect on overall mental well-being and mindfulness with no specific mention of suicidality. Except Larsen et al [13], the apps in other studies were developed to address symptoms of mental disorders related to suicidality. These symptoms included depression and anxiety. In summary, the extent of generalizability of such findings to suicidality in Asian youths remains questionable.

The research reviewed in Multimedia Appendix 1 indicated that considerations for future research should include interventions lasting more than 10 days that had more than one postintervention measurement [76]. To reduce drop-out rates, reminders should be sent to users [69]. Researchers should carefully consider power and sample size and ensure robustness in statistical analyses.

There is currently a lack of interactive self-care apps available to Asian users that incorporate explicit delineation of the scope or initial screening for suitability, or offering targeted guidance, regarding management of suicidal crises [74]. Few of the apps currently on the market included content aimed at encouraging professional help-seeking or had an explicit mention of the theoretical or empirical basis of interventions. This gap needs to be addressed by partnerships between scholars, software engineers, and specialists in biomedical informatics to develop, test, and refine appropriate interfaces and apps. When designing such a mindfulness app, features to be considered include: the evidence base supporting use of mindfulness techniques in Asia; and the consideration of all the aforementioned issues, such as inclusion of explicit delineation of the scope or initial screening for suitability, targeted guidance, linking users with professional help-seeking, or explicit mention of the theoretical or empirical basis of mindfulness interventions. Specifically, mindfulness features in the app may include: breathing, body scanning, sitting meditations, walking meditations, loving kindness meditations, thoughts and emotion focus, mountain meditation, lake meditation, and 3-minute breathing spaces [53]. The content of apps for suicidality should contain at least one interactive suicide prevention feature (eg, safety planning, facilitating access to crisis support) and contain at least one strategy consistent with the evidence base or relevant best-practice guidelines [13]. Potentially harmful content, such as listing lethal access to means or encouraging risky behavior in a crisis, should be carefully screened and eliminated. Psychoeducational components to reduce the stigma related to suicidality and mental illnesses could be incorporated [44], together with monitoring of moods and stressors or other suicide triggers [45]. Youths are adversely affected by many psychosocial stressors, such as interpersonal stress which triggers suicidal ideation [78]; such triggers should be carefully assessed and addressed [4].

Another consideration is that suicidal Asian youths are not a homogenous group [4]. Suicide risk assessment needs to be conducted with consideration of risk and protective factors [45]. Therapeutic needs must be considered before clinicians decide on suitability for the use of a mindfulness app with their patients. Clinicians should carefully examine the prevailing code of ethics in working with suicidal clients to ensure best practices are observed [4,44]. This approach may include a comprehensive suicide risk assessment before deciding on the best intervention for the client [45]. Other factors to consider include defining the primary therapeutic goal and outcome (eg, reduced intensity or frequency of suicidal ideation, reduced lethality [4,44], or reduced frequency of repeated suicide attempts [45]) and monitoring the therapeutic gains progressively. It is unclear if suicide risk screening and monitoring using a smartphone app could replace face-to-face assessment conducted by an experienced clinician, but the prevailing code of ethics and professional best practices do not currently support this [4,44,45], especially when the evidence base is not clearly demonstrated.

A limitation of the review stems from the inconsistencies of the study types included in the review. Narrative reviews were included to inform the context but should be excluded because they are challenging to compare across study types. This issue further highlights the paucity of research in this area. Future research could focus on empirical studies and randomized controlled trials with Asian samples that conform to CONSORT guidelines [79]. Further research is also needed to examine the parametrization of the characteristics of the apps and their quantitative analysis with Asian samples. In addition, it is unclear if discrepancies exist between Asian samples from developing and developed countries, which could be explored in future research. The strengths of the review include the investigation of an important clinical issue and highlighting the need for more research on this pertinent topic.

In conclusion, there is consensus that suicidal risk in youths is a rising concern, especially in Asia in recent years [4]. The potential use of smartphone apps in the delivery of mindfulness interventions tailored for suicidality in Asian youths remains promising, but the evidence base to support their use is lacking. More research is needed to address the current gaps in knowledge and to provide an evidence base for the implementation of smartphone technologies. Developing mobile tools for young suicidal users requires careful ethical consideration regarding the patient-practitioner relationship, the logic of self-surveillance, prevailing codes of ethics, and overall best practices. More rigorous research and evaluations are needed to ascertain the efficacy of, and establish evidence for, best practices for the usage of such smartphone apps [40].

## Appendix

Multimedia Appendix 1 Summary of evidence.
