# Supplementary material for: Smartphone Apps for Mindfulness Interventions for Suicidality in Asian Youths: Literature Review
Source: JMIR Mhealth Uhealth. 2018 Jun 7;6(6):e139. doi: 10.2196/mhealth.8304 (PMC6013712; doi:10.2196/mhealth.8304)
Supplement: Multimedia Appendix 1 [file mhealth_v6i6e139_app1.pdf]

Multimedia Appendix 1. Summary of evidence.

Author/Year	Level of Evidence/Study Design/Participants/ Inclusion Criteria	Outcome Measures/ Study objectives	Results
Aggarwal (2012)	Level IV<br><br>Narrative review	The study aimed to review the use of smartphone applications in South Asia.	There is a shortage of mental health professionals in South Asia, yet a high penetration of mobile phones throughout South Asia.
Brian & Ben-Zeev (2014)	Level IV<br><br>Narrative Review	Primary objective of the study was to review the reach of mobile technology in Asia, and consider the integration of smartphone applications into the study, diagnoses, and treatment of mental disorders in Asia	Asian countries have smartphone usage over 50% of the populations, with several countries (e.g. Maldives, Singapore and Vietnam) reporting smartphone adoption rates that far exceed the number of people in the population.
Cavanagh, Strauss, Forder & Jones (2014)	Level I<br><br>Systematic review and meta-analysis<br><br>Fifteen Randomized Controlled Trials (including 4 mindfulness-based interventions) were identified and reviewed.	The aim of the study was to evaluate the effectiveness and acceptability of low-intensity interventions with mindfulness features or acceptance components. Primary outcomes included measures of mindfulness, depression and anxiety.	Interventions that included mindfulness and/or acceptance-based components produced significant benefits in comparison to control conditions on measures of mindfulness/acceptance, depression and anxiety with small to medium effect sizes.<br><br>Engagement with the self-help interventions varied but on average

			two-thirds of participants completed post-intervention measures.<br><br>There were high drop-out rates and few trials were adequately powered.
Mak, Chan, Cheung, Li & Ngai (2015)	Level I<br><br>Randomized controlled trial<br><br>N=321 university students and staff<br><br>(Chinese ethnicity, Mean age = 24 years)	The aim of the study was to evaluate the efficacy of Internet-based mindfulness interventions that can be accessed by digital interface platforms such as smartphones and tablets.	The mindfulness intervention had a significant effect on overall mental well-being (*p*=.02) and mindfulness (*p*=.27)
Mani, Kavanagh, Hides & Stoyanov (2015)	Level I<br><br>Systematic Review<br><br>23 applications reviewed	The study aimed to conduct a systematic review of current mindfulness based smartphone applications for mental health and to evaluate their quality and efficacy	Of the 700 applications that were initially screened, 23 applications met the inclusion criteria and were reviewed.<br><br>Main features across mindfulness based applications include: breathing, body scanning, sitting meditations, walking meditations, loving kindness meditations, thoughts and emotion focus, mountain meditation, lake meditation and three

			minute breathing spaces.
Hoswells, Iytzan & Eiroa-Orosa (2016)	Level I<br><br>Randomized Control Trial<br><br>*N*=194<br><br>97 participants were assigned to the experimental condition and 97 to the control condition.<br><br>Only 1.7% of the sample was Asian.	Objective was to measure the efficacy of a mindfulness based smartphone application designed to enhance well-being	The findings on the mindfulness meditation application 'Headspace' found that it significantly increased positive affect (p=.003) and decreased depression (p=.05). No statistically significant difference in satisfaction with life or negative affect was found and may be attributable to the limited time duration of the research (intervention only lasted for 10 days) and there was only one post-intervention measurement.
Larsen, Nicholas & Christensen (2016)	Level IV<br><br>123 applications referring to suicide were reviewed	The primary objective of the study was to review the content of publicly available applications for suicide prevention.	49 applications contained at least one interactive suicide prevention feature. Most applications focused on obtaining support from friends and family (n = 27) and safety planning (n = 14). Of the different suicide prevention strategies contained within the applications, the strongest evidence in the literature was found for facilitating access to crisis support (n = 13). All reviewed

			contained at least one strategy that was broadly consistent with the evidence base or best-practice guidelines. Applications tended to focus on a single suicide prevention strategy although safety planning incorporated a greater number of techniques (mean = 3.9). Potentially harmful content, such as listing lethal access to means or encouraging risky behaviour in a crisis, was also identified.
Spijkerman, Pots & Bohlmeijer (2016)	Level I<br><br>Review and meta-analysis<br><br>Fifteen randomised controlled trials were included in this study.<br><br>Representation of Asian populations was limited. (Only a small fraction less than 15% were of Asian ethnicity)	The meta-analysis aimed to examine the effects of online Mindfulness Based Interventions on mental health	The results found that online interventions are a promising strategy to alleviate psychological symptoms and reduce the prevalence of severe mental health problems.<br><br>Across the studies, the mindfulness based interventions were found to have significant effects on well-being ($p<.001$), stress ($p<.001$), anxiety ($p=.010$), depression ($p<.001$) and mindfulness ($p<.001$)
Garcia, Sanchez,	Level II	The aim of the study	The applications

Espilez, Magarino, Guillen & Garcia-Campayo (2017)	*N*=3,951<br><br>3977 users were involved in this study: 26 in the first trial during an 8-week usage period and 3951 in the second trial during 17 months<br><br>(7.7% of the sample were below the age of 29 years)	was to examine a first prototype of a smartphone application with Spanish features for the training and practice of mindfulness. The outcome measures were acceptance and perceived quality of the application as well as data about the usage	'Mindfulness' and 'Mindfulness Sci' were designed in consultation with licensed health professionals. 3,951 people downloaded the application. In the first study, participants assessed the application and considered it as a helping tool for mindfulness practice, and user-friendliness. In the second study, weak associations were found between usage time and age, nationality and educational level. The mindful level showed a weak positive correlation with the session accomplished (p = 0.051). Videos and information stood out as the most accessed resources.
Kumar & Mehrotra (2017)	Level I<br><br>Review Paper<br><br>33 interactive self-care smartphone applications were reviewed	Primary objective was to identify the mindfulness related mobile applications available to Indian android phone users	Information on coping with depression screening tools formed the two largest types of free applications. Interactive self-care applications were reviewed further and less than 10% of the applications incorporated explicit delineation of their scope or initial screening for suitability. Guidance regarding managing suicidal crisis were incorporated in only

			about 12% of the interactive applications. About one third of these included content aimed at encouraging professional help seeking or had an explicit mention of the theoretical or empirical basis. Mindfulness techniques, monitoring moods, thoughts and behaviors were the most common therapeutic strategies incorporated in these applications, namely behavioral activation, identifying and correcting cognitive errors, cultivation of gratitude, and medication management.
